# Tim Swanwick, Understanding medical education: evidence, theory and practice: Wiley-Blackwell, 2013, 520 pp., 2nd edn, ISBN 978-1-118-47240-8

**DOI:** 10.1007/s40037-014-0113-4

**Published:** 2014-03-04

**Authors:** Griet Peeraer

**Affiliations:** Faculty of Medicine and Health Sciences, University of Antwerp, Universiteitsplein 1 S032, 2610 Wilrijk, Belgium

 ‘Understanding medical education is a synopsis of educational theory and practice that is easily navigated and covers a variety of topics and themes that are crucial in health professions organization’ (foreword to the second edition, p. 11).

The first sentence of the second edition describes its content accurately. The book is divided into five ‘parts’: foundations, educational strategies, assessment, research and evaluation, staff and students. Each part holds a number of topics, relevant to health education. For each topic (chapter), experts were invited to share their knowledge/research and also recent developments in their field of expertise. The second edition of this book offers updates on more than 30 topics and includes some new chapters on topics that have come to light in contemporary medical education.

‘Understanding medical education’ is accessible for students and professionals who already have some experience in medical education but wish to deepen their knowledge. As all authors refer to scientific publications and offer literature for further reading, the book can be a gateway to gathering more in-depth knowledge. But it is also a useful book for new colleagues. The first two chapters (Understanding medical education, Teaching and learning in medical education: how theory can inform practice) should be compulsory literature for all teachers entering the field of medical education. Staff development organizers may benefit from this book as a source of reading material.

The title of the book suggests that evidence, theory and practice are offered. This is the case: special icons refer to scientific evidence, to a special focus on a topic, to the transfer from theory to practice and to the key messages. Unfortunately not all chapters use these icons, whereas especially the ‘how to’ (transfer from theory to practice) comes in very handy for new professionals in medical education. Chapters that use all or most of the icons are of a more practical nature than others.

Ara Tekian states in the book’s foreword: ‘*the single best companion* for medical educators, health professionals, physicians, nurses, allied health professionals, programme directors, health practitioners, and students in certificate and Masters’ programmes in health professions education’.

PME’s final verdict: not the single best, but *one of the vital companions* for all professionals in the field of medical education.

